# Supplementary material for: A data-driven approach for timescale decomposition of biochemical reaction networks
Source: mSystems. 2024 Jan 23;9(2):e01001-23. doi: 10.1128/msystems.01001-23 (PMC10946255; doi:10.1128/msystems.01001-23)
Supplement: Supplemental figures — Fig. S1-S4. [file msystems.01001-23-s0003.pdf]

Supplementary Materials for

A data-driven approach for timescale decomposition of biochemical reaction networks

Amir Akbari[1], Zachary B. Haiman[1], Bernhard O. Palsson[1,2,*]

[1] *Department of Bioengineering, University of California San Diego, La Jolla, CA 92093*
[2] *Novo Nordisk Foundation Center for Biosustainability, Technical University of Denmark, 2800 Lyngby, Denmark*

This PDF file includes:
Figs. S1–S4

[*] Corresponding author.
Email addresses: amakbari@ucsd.edu (Amir Akbari), zhaiman@ucsd.edu (Zachary B. Haiman), palsson@ucsd.edu (Bernhard O. Palsson)

A

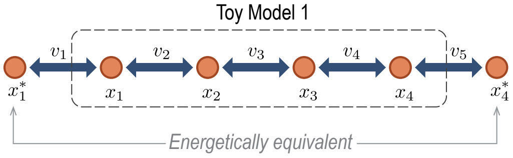

B

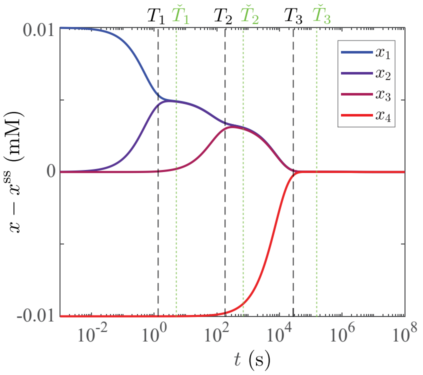

C

Reactions	k^+ (1/s)	K^{eq}
1	10^{-6}	1
2	10^{0}	1
3	10^{-2}	1
4	10^{-4}	1
5	10^{-6}	1

Modes	λ^{ss} (1/s)	τ (s)
1	-2.0×10^{0}	5.0×10^{-1}
2	-1.5×10^{-2}	6.7×10^{1}
3	-1.3×10^{-4}	7.5×10^{3}
4	-5.0×10^{-7}	2.0×10^{6}

Figure S1: A toy model without energy coupling, where the substrate (Metabolite 1) and product (Metabolite 4) are energetically equivalent. (A) Network map. (B) Dynamic response to concentration perturbations with four characteristic timescales. (C) Rate constants and timescales. The superscript * indicates metabolite concentration in the extracellular environment.

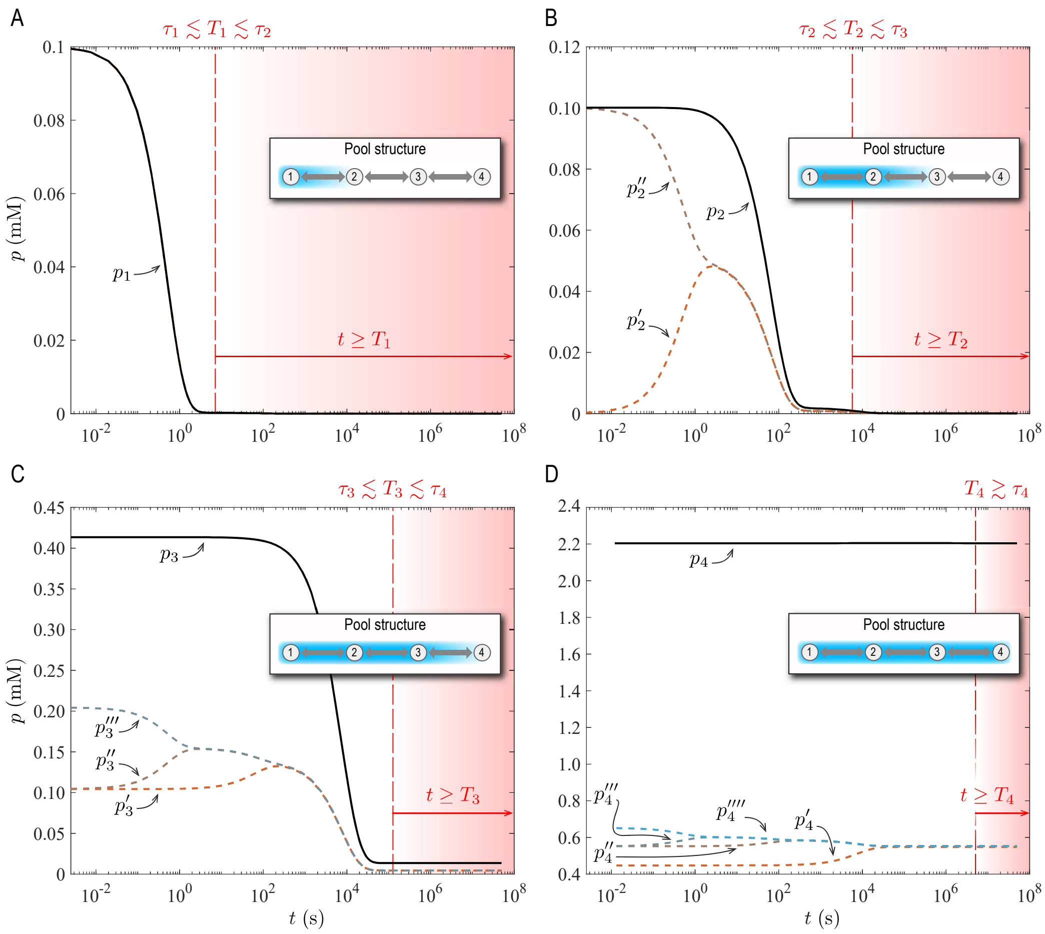

Figure S2: Pool representations for Toy Model 1 described in Fig. 2. (A) Representation of Pool 1 associated with the disequilibrium of Reaction 2. (B) Representations of Pool 2 associated with the disequilibrium of Reaction 3. (C) Representations of Pool 3 associated with the disequilibrium of Reaction 4. (D) Representations of Pool 4 associated with the conservation of all the intracellular metabolites. Timescales $\tau_{1\text{–}4}$ are provided in Fig. 2B. The fourth timescale is defined as $T_4 := \check{T}_3$ (see Fig. 2B), where $\check{T}_3$ is a timescale characterizing a transitory regime between the third timescale and steady state of Toy Model 1.

A

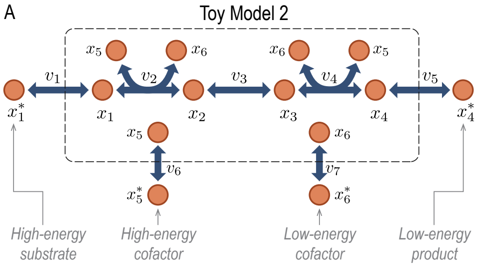

B

Reactions	k^+ (mM^{1-d}/s)	K^{eq}	d
1	10^{-3}	1	1
2	10^{0}	1	2
3	10^{-2}	1	1
4	10^{0}	1	2
5	10^{-3}	1	1
6	10^{0}	1	1
7	10^{0}	1	1

Modes	λ^{ss} $(1/\mathrm{s})$	τ (s)
1	-2.3×10^{0}	4.3×10^{-1}
2	-1.0×10^{0}	1.0×10^{0}
3	-3.8×10^{-1}	2.6×10^{0}
4	-1.4×10^{-1}	7.0×10^{0}
5	-1.2×10^{-2}	8.5×10^{1}
6	-4.3×10^{-4}	2.3×10^{3}

d: Degree of forward reaction

$\varkappa$: Reference concentration in mM

$$\frac{k_j^+}{k_j^-} = \varkappa^{\sum_{i=1}^{n} S_{ij}} K_j^{\mathrm{eq}}$$

Figure S3: A toy model with energy coupling, where a high-energy substrate (Metabolite 1) is converted to a low-energy product (Metabolite 4). The energy released in the main pathway fuels the production a high-energy cofactor (Metabolite 5) from its low-energy counterpart (Metabolite 6). (A) Network map. (B) Rate constants and timescales. The superscript * indicates metabolite concentration in the extracellular environment.

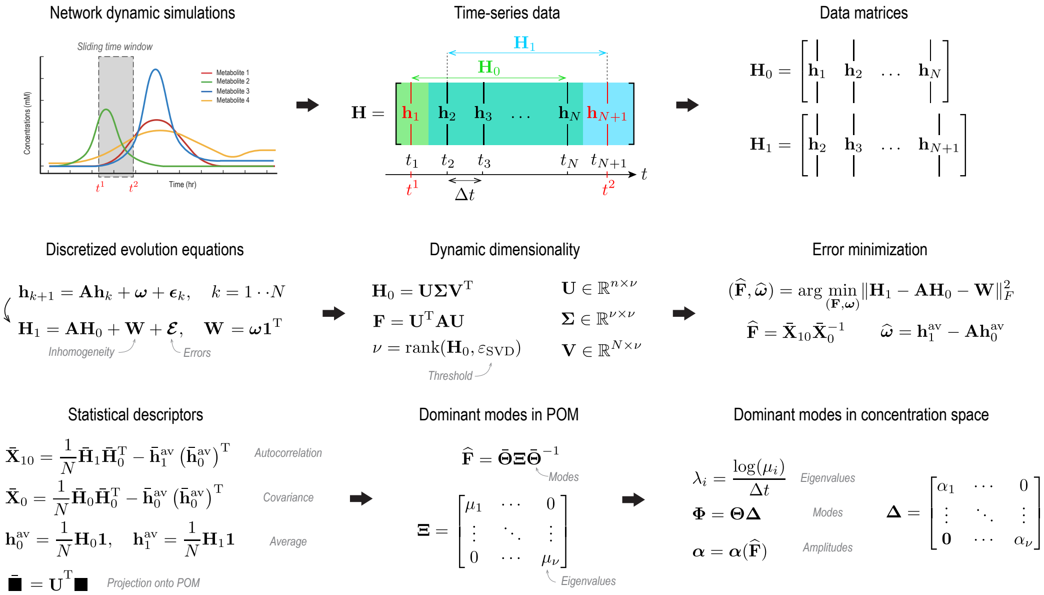

Figure S4: An extended version of Optimal Dynamic Mode Decomposition applied in Dynamic Mode Analysis to identify dominant eigenmodes and eigenvalues locally from time-series data. The space of proper orthogonal modes is denoted POM.
